# The MYCN-HMGA2-CDKN2A pathway in non-small cell lung carcinoma—differences in histological subtypes

**DOI:** 10.1186/s12885-016-2104-9

**Published:** 2016-02-08

**Authors:** Hanne A. Eide, Ann Rita Halvorsen, Maria Moksnes Bjaanæs, Hossein Piri, Ruth Holm, Steinar Solberg, Lars Jørgensen, Odd Terje Brustugun, Cecilie Essholt Kiserud, Åslaug Helland

**Affiliations:** Department of Cancer Genetics, Institute for Cancer Research, Oslo University Hospital-The Norwegian Radium Hospital, Oslo, Norway; Department of Oncology, Oslo University Hospital-The Norwegian Radium Hospital, Oslo, Norway; Cellular and Molecular Research Center, Qazvin University of Medical Sciences, Qazvin, Iran; Department of Pathology, Oslo University Hospital-The Norwegian Radium Hospital, Oslo, Norway; Department of Cardiothoracic Surgery, Oslo University Hospital-Rikshospitalet, Oslo, Norway; Department of Oncology, National Advisory Unit on Late Effects After Cancer Treatment, Oslo University Hospital-The Norwegian Radium Hospital, Oslo, Norway

**Keywords:** Lung cancer, NSCLC, *HMGA2*, *MYCN*, *CDKN2A*, *DICER1*, *Let-7*, survival

## Abstract

**Background:**

Extensive research has increased our understanding of the molecular alterations needed for non-small cell lung cancer (NSCLC) development. Deregulation of a pathway including MYCN, HMGA2 and CDKN2A, with the participation of DICER1, is of importance in several solid tumours, and may also be of significance in the pathogenesis of NSCLC.

**Methods:**

Gene expression of *MYCN*, *HMGA2*, *CDKN2A* and *DICER1* were investigated with RT-qPCR in surgically resected NSCLC tumour tissue from 175 patients. Expression of the let-7 microRNA family was performed in 78 adenocarcinomas and 16 matching normal lung tissue samples using microarrays. The protein levels of HMGA2 were determined by immunohistochemistry in 156 tumour samples and the protein expression was correlated with gene expression. Associations between clinical data, including time to recurrence, and expression of mRNA, protein and microRNAs were analysed.

**Results:**

Compared to adenocarcinomas, squamous cell carcinomas had a median 5-fold increase in mRNA expression of *HMGA2* (*p* = 0.003). A positive correlation (*r* = 0.513, *p* < 0.010) between *HMGA2* mRNA expression and HMGA2 protein expression was seen. At the protein level, 90 % of the squamous cell carcinomas expressed high levels of the HMGA2 protein compared to 47 % of the adenocarcinomas (*p* < 0.0001). *MYCN* was positively correlated with *HMGA2* (*p* < 0.010) and *DICER1* mRNA expression (*p* < 0.010), and the expression of the let-7 microRNAs seemed to be correlated with the genes studied. *MYCN* expression was associated with time to recurrence in multivariate survival analyses (*p* = 0.020).

**Conclusions:**

A significant difference in *HMGA2* mRNA expression between the histological subtypes of NSCLC was seen with a higher expression in the squamous cell carcinomas. This was also found at the protein level, and we found a good correlation between the mRNA and the protein expression of HMGA2. Moreover, the expression of *MYCN*, *HMGA2*, and *DICER1* seems to be correlated to each other and the expression of the *let7*-genes impacted by their expression. *MYCN* gene expression seems to be of importance in time to recurrence in this patient cohort with resected NSCLC.

**Electronic supplementary material:**

The online version of this article (doi:10.1186/s12885-016-2104-9) contains supplementary material, which is available to authorized users.

## Background

The prognosis of lung cancer is dismal. For all stages, 5 year relative survival are 19 % for women and 14 % for men in Norway [1]. Worldwide, lung cancer is the most common cause of cancer related death and it is estimated that 1.6 million people die annually due to lung cancer [2].

Non-small cell lung cancer (NSCLC) constitutes over 80 % of all lung cancers and can be divided into histological subtypes. Investigation to further unveil the NSCLC biology includes molecular characterization. Epidermal growth factor receptor (*EGFR*) mutational testing has been performed routinely in Norway since 2010. Targeted therapies such as EGFR inhibitors and anaplastic lymphoma kinase (ALK) inhibitors are currently in clinical use. Increased understanding of different levels of tumour development, of genetic, epigenetic, protein alterations and their functional influence are of clinical importance. For a disease where a majority of patients are diagnosed in late stages, there is a need to discover biomarkers as well as new targets for therapeutic interventions.

Overexpression of the *HMGA2* gene is linked to the development of cancer [3]. The *HMGA2* gene encodes a non-histone chromatin modifying protein that binds to AT-rich regions in the DNA; thus leading to a change in DNA structure and interaction with transcription factors influencing cell growth, proliferation, differentiation and cell death [4]. HMGA proteins are abundantly expressed during fetal development, but scarcely present or even absent in normal adult tissue [5]. In NSCLC, however, re-expression of HMGA2 is proposed as a common event and considered as a molecular marker [6]. Indeed, HMGA2 protein expression is in some studies shown related to lung cancer development and progression, and inversely associated with lung cancer survival [7, 8].

The *MYC* oncogenes are well characterized participants in cell proliferation, growth, differentiation and apoptosis [9]. One of the MYC family members, MYCN, functions as a positive regulator of LIN28B, a known repressor of the let-7 family of microRNAs [10, 11]. MicroRNAs are small non-coding RNAs that regulate gene expression, making them important players in cancer development and progression [12]. LIN28 protein binds to pre-let-7 s and prevents further processing into mature let-7 microRNAs by DICER1 [13]. HMGA2 is a thoroughly described target of let-7 s and a loss of let-7 microRNAs can lead to HMGA2 overexpression, shown both in cell lines and in solid tumours [14, 15]. Furthermore, through downregulation of the tumour suppressor gene *CDKN2A*, HMGA2 is shown to be involved in stem cell renewal [16]. CDKN2A level is previously demonstrated as an independent prognostic factor in non-small cell lung carcinoma [17].

**Fig. 1:**
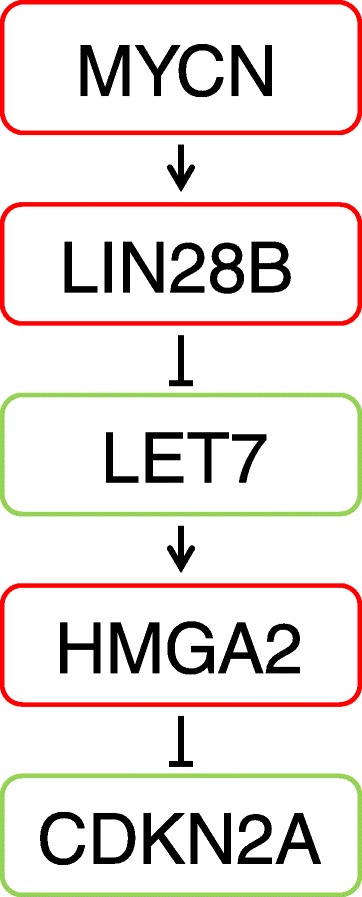
The MYCN, HMGA2, CDKN2A pathway. MYCN functions as a positive regulator of LIN28B, a known repressor of the let-7 family of microRNAs *via* binding to DICER1. Loss of let-7 microRNAs can lead to HMGA2 overexpression that may result in a downregulation of CDKN2A

The role of a deregulated pathway including MYCN, HMGA2 and CDKN2A with the participation of DICER1 (Fig. 1) has not previously been investigated in NSCLC. In this study we explore the significance of *MYCN*, *HMGA2*, *CDKN2A* and *DICER1* in NSCLC tumours by gene expression analysis. HMGA2 protein expression data are evaluated as well as microarray information on let-7 microRNAs, to elucidate possible correlations. Differences in histological or clinical subgroups are assessed. Finally, associations with time to recurrence are examined.

## Methods

### Patient cohort

Eligibility criteria for the present study were patients diagnosed with lung cancer stage I-IIIa, treated with curatively intended surgery at Oslo University Hospital – Rikshospitalet, from 2006 to 2010.

Clinical data were collected from the hospital’s medical records. Follow up data procured via questionnaires administered to the patients, through copies of medical records from local hospitals and information from general practitioners. All data were registered in a project database. The patients were followed until death or 5 years past surgery. Tumours were staged according to the Union for International Cancer Control (UICC), TNM 7, and histopathological parameters were retrieved from the pathology reports. Tumours were grouped in three based on histology; adenocarcinomas, squamous cell carcinomas and others (including large cell carcinomas, undifferentiated carcinomas, adeno-squamous carcinomas, small cell carcinomas and carcinoids). Neoadjuvant and adjuvant treatment were given according to guidelines based on TNM stage and the patient’s age.

### Tumour samples

The tumour specimens were collected by thoracic surgeons during lung cancer surgery. They were snap-frozen in liquid nitrogen and stored at − 80 °C until further processing.

### RNA isolation

Total RNA was extracted from the tumour specimens using standard TRIZOL methods (Invitrogen, Carlsbad, CA). RNA quantity and quality were assessed by NanoDrop ND-1000 spectrometer (NanoDrop technologies) and by RNA integrity numbers (RIN) measured using the 2100 Bioanalyzer (Agilent Technologies, Santa Clara, CA).

### Reverse transcription quantitative polymerase chain reaction (RT-qPCR) for gene expression

Gene expression of the four selected genes; *MYCN*, *HMGA2*, *CDKN2A* and *DICER1* was measured on tumour samples by RT-qPCR using the Applied Biosystems 7900HT Fast Real-Time PCR system (7900HT Fast System, Applied Biosystems, Foster City, CA). Single stranded cDNA was synthesized from totalRNA in samples using High-Capacity cDNA Reverse Transcription Kit (Applied Biosystems). Human *ACTB* (beta actin) was used as endogenous control and a commercial Ambion breast control (Life Technologies) used as a calibrator in the qPCR. *ACTB* where chosen as a single endogenous control when the RT-qPCR where performed in 2011–2012 based on earlier satisfactory experience at our institution, and the current perception of endogenous controls in RT-qPCR experiments in general. All reactions were performed as triplicates for each sample. Outliers were omitted from further analysis. Negative and positive controls were included in every run.

The mRNA expression values was quantified as ΔΔCt values, where Ct = threshold cycle, ΔCt = (Ct target mRNA - Ct B-actin) and ΔΔCt = (ΔCt target mRNA - ΔCt calibrator). Relative quantification (fold change) of mRNA expression was calculated by the 2 ^–ΔΔCt^ method [18] using the RQ manager software, version 1.2 (Applied Biosystems).

In a total of 175 patients included, gene expression data was generated from 170 tumour samples for *MYCN*, 132 tumour samples for *HMGA2*, 171 tumour samples for *CDKN2A* and 164 tumour samples for *DICER1*, respectively. A REMARK diagram of the patients is presented in Additional file 1: Figure S1.

Additional RT-qPCR analyses did not pass the quality control or were not possible to perform due to lack of sufficient tumour material.

### Immunohistochemistry

HMGA2 protein expression levels in the tumour tissue were determined by immunohistochemistry. We used rabbit anti-HMGA2 antibodies (www.biocheckinc.com) and Dako EnVision Flex + System (K8012) on tissue micro arrays (TMAs) from 156 tumour samples. All samples were represented on the TMAs in duplicates. Protein expression was scored by counting nuclear staining positivity; either as negative (0), less than 10 % tumour cells with strong nuclear staining (1+), 10–50 % tumour cells with strong nuclear staining (2+) or more than 50 % tumour cells with strong nuclear staining (3+). Samples with score 2+ and 3+ were considered overexpressing the HMGA2 protein.

### MicroRNA expression

For microRNA profiling, microRNA microarrays from Agilent Technologies (Agilent human microRNA microarray kit release 16.0, 8 x 60 K) was used in 78 tumour tissue samples. For 16 of the cases matching normal lung tissue was also available for analysis. The normal lung tissue was collected from the lung or lobe removed during the operation, at least 10 cm apart from the macroscopic tumour. The microRNA kit encodes 1205 human microRNAs and 144 human viral microRNAs listed in the Sanger miRBase (release 16.0). Arrays were scanned with Agilent Microarray Scanner (Agilent Technologies, Santa Clara, CA) and raw data pre-processed with Agilent Feature Extraction Software (v. 10.7.3.1), with default parameters employed. MicroRNAs detected in less than 10 % of the samples were filtered out, 570 microRNAs, including 10 microRNAs of the let-7family, remained for further analysis. Microarray data were processed by log_2_ transformation, and normalized between arrays by the 90^th^ percentile method using the Genespring GX analysis Software v.12.1 (Agilent Technology). Some of the results on the microRNA profiling have previously been published by Bjaanæs et al. [19] and characteristics of the patients are presented in Additional file 1: Table S1.

### Validation

For validation of the survival analyses, two data sets were used. The first set contained data from mainly adenocarcinomas from our own research group, while the other was composed of data from squamous cell carcinomas obtained *via* the The Cancer Genome Atlas (TCGA) data portal [20].

We used the SurePrint G3 Human Gene Expression 8x60K Microarray Kit (Agilent Technologies) for mRNA profiling in 187 lung cancer samples, including 184 lung adenocarcinomas. The arrays were scanned with Agilent C scanner (Agilent’s Scan control software, version A.8.4.1) and the dataset extracted with the Agilent Feature Extraction Software. In GeneSpring GX 12 (Agilent Technologies) the data was normalized by using the 75 percentile method. Median follow up for patients still alive in this cohort who had not developed metastasis or a local recurrence was 49 months (range 26–60 months). Forty percent of the patients in this cohort had a recurrence of lung cancer disease during the follow-up time.

A dataset of lung squamous cell carcinoma was obtained through the TCGA data portal where mRNA expression data were available in a total of 508 tumour specimens defined as lung squamous cell carcinoma. Clinical data and details of progression and survival were, however, only available for a total of 280 of the patients on the website, and these patients were thus included in the survival analysis. Median follow up time in the TCGA dataset for patients still alive with no metastasis or local recurrence was 20 months, ranging from 2 to 60 months and 27 % of the patients experienced local recurrence or distant metastasis during follow-up.

Clinical details for both datasets are available in Additional file 1: Table S2 and Additional file 1: Table S3.

### Statistical analyses

Data are reported using descriptive statistics with percentages, means, standard deviations, medians and ranges.

Relative quantification of mRNA expression (fold change) of the selected genes; *MYCN*, *HMGA2*, *CDKN2A* and *DICER1* in the tumour samples was analysed in different clinical subsets such as histology, stage, sex and smoking history. Differences in median values between groups regarding continuous mRNA expression data were analysed using non-parametric tests, Mann–Whitney *U* test and/or Kruskal Wallis test where appropriate. Fold change values are not normally distributed and analyses of correlations were done with Spearman’s Rank Order correlation. Expression of HMGA2 protein in different histological entities, were analyzed by Chi-square test. To evaluate differences in mean let-7 microRNA expression in tumour and normal lung tissue, and to see whether mRNA expression were associated with mean microRNA let-7 data, independent t-tests were performed.

For the survival analyses, follow up data existed for all included patients. Time to recurrence of lung cancer was calculated as the interval between the date of operation and date of local relapse, distant metastases, or death due to lung cancer. Patients that died of other causes without local relapse or metastases were censored in the survival analyses (*n* = 38). One of the participants died 13 days after the operation in cardiac arrest, and was excluded from the survival analyses. Of the remaining 37 patients that died of alternative causes it was verified through medical records that 15 patients died from cardiovascular disease. 15 patients died from other verified diseases such as COPD, pneumonia and septicaemia. For 8 patients no definitive cause of death were uncovered, however neither of these patients had a verified relapse of NSCLC prior to death, nor were there any indication of a lung cancer related death in the medical records procured.

Survival curves and estimation of statistical significance between groups were performed using the Kaplan-Meier method and log rank test, respectively; here mRNA expression (fold change) was categorized into high and low values based on the median gene expression value. Factors associated with lung cancer progression were analyzed using the Cox proportional hazard regression model also using dichotomous fold change data for gene expression. Although not significant in the log rank tests performed, age at surgery, sex, stage and histology, were still included in the multivariate Cox regression analysis, due to high clinical relevance.

A *p*-value ≤ 0.050 was considered statistically significant. All statistical analyses were performed using SPSS version 21.0 (SPSS Inc., Chicago, MO, USA).

### Ethical considerations

This project was approved by the regional ethics committee (Regional comittees for medical and health research ethics - South East) (Approval no:S-06402b) and the institutional review board (Protokollutvalget, Radiumhospitalet). The patients were given oral and written information prior to inclusion. A written consent was obtained from all the participants.

## Results

### Patient characteristics

One hundred and seventy-five patients were included in the study. The median age at surgery was 66 years (range 34 to 82 years) and 54 % were male. The tumour samples included 57 % adenocarcinomas, 25 % squamous cell carcinomas and 18 % others. According to the current TNM classification, 55 % of the patients were in pTNM stage I, 31 % in stage II and 13 % in stage IIIa. Fifty four of the patients (31 %) received adjuvant chemotherapy, 3 patients received neoadjuvant chemotherapy and 9 patients received radiotherapy (adjuvant or neoadjuvant). During follow up, 60 participants (34 %) presented with relapse of disease; either as a local recurrence or metastatic disease. At the end of follow up 86 patients (49 %) had died. For those whose disease recurred, the median time from initial surgery to diagnosis of relapse was 18 months (range 2 to 60 months). Clinical and pathological features and outcome parameters are summarized in Table 1.

**Table 1:** Characteristics of the patient cohort

Age at surgery (y):		
Median/range:	66/34–82	
	*n* = 175	%
Sex:		
Male:	95	54.3
Female:	80	45.7
Smoking history:		
Current:	107	61.1
Former:	57	32.6
Never:	11	6.3
Weight loss:		
No weight loss:	48	27.4
Weight loss < 10 %	99	56.6
Weight loss ≥ 10 %	22	12.6
No data:	6	3.4
Tumor size:		
< 3 cm	99	56.6
> 3 cm - ≤5 cm	55	31.4
> 5 cm- ≤7 cm	19	10.9
> 7 cm	2	1.1
Stage:		
I:	97	55.4
II:	55	31.4
IIIa:	23	13.1
Histology:		
Adenocarcinoma:	99	56.6
Squamous cell carcinoma:	44	25.1
Other:	32	18.3
EGFR:		
EGFR mutated:	13	7.4
EGFR not mutated:	159	90.9
EGFR not tested:	3	1.7
Chemotherapy:		
Adjuvant chemotherapy:	54	30.9
Neoadjuvant chemotherapy:	3	1.7
No chemotherapy:	116	66.3
No data:	2	1.1
Radiotherapy:		
Adjuvant or neoadjuvant:	9	5.1
No radiotherapy	166	94.9
Status at 5 year follow up:		
Alive with no disease:	81	46.3
Alive with disease:	8	4.6
Dead:	86	49.1
Cause of death (*n* = 86):		
Lung cancer:	48	55.8
Cardiovascular disease:	15	17.4
Other:	23	26.7

### Gene expression

We discovered a significant difference in the median values of *HMGA2* mRNA expression according to tumour histology (*p* = 0.003) with a considerably higher median fold change value in the squamous cell carcinoma group (70.6 fold) compared to adenocarcinomas (13.1 fold) and others (5.8 fold) (Table 2). Regarding *CDKN2A* expression, a significantly higher median expression was found in women compared to men (*p* = 0.013). Boxplots illustrating the distribution of gene expression is presented in Additional file 1: Figure S2.

**Table 2:** Relative quantification of mRNA expression (fold change) in tumour sample subsets stratified by histology, stage, sex and smoking history

Genes	All	Histology				Stage				Sex			Smoking history			
		AC	SCC	Other	*p*-value	I	II	III	*p*-value	Male	Female	*p*-value	Never	Former	Current	*p*-value
MYCN	*n* = 170	*n* = 94	*n* = 44	*n* = 32		*n* = 94	*n* = 54	*n* = 22		*n* = 91	*n* = 79		*n* = 11	*n* = 54	*n* = 105	
median	0.43	0.46	0.54	0.24	0.517	0.46	0.38	0.40	0.723	0.47	0.37	0.057	1.12	0.47	0.37	0.195
(range)	(0.01–943.85)	(0.03–943.85)	(0.02–10.25)	(0.01–43.00)		(0.02–88.53)	(0.01–166.37)	(0.32–943.85)		(0.01–943.85)	(0.03–166.37)		(0.20–13.55)	(0.01–88.53)	(0.03–943.85)	
HMGA2	*n* = 132	*n* = 71	*n* = 36	*n* = 25		*n* = 70	*n* = 41	*n* = 21		*n* = 79	*n* = 53		*n* = 8	*n* = 44	*n* = 80	
median	20.01	13.12	70.65	5.77	0.003**	15.33	40.88	7.58	0.360	17.33	23.16	0.758	18.28	29.14	16.98	0.945
(range)	(0.21–33877.07)	(0.56–2736.65)	(0.71–33877.07)	(0.21–10749.96)		(0.21–13499.32)	(0.27–33877.07)	(0.31–2168.39)		(0.27–33877.07)	(0.21–13499.32)		(2.67–640.82)	(0.21–13499.33)	(0.31–33877.07)	
CDKN2A	*n* = 171	*n* = 96	*n* = 44	*n* = 31		*n* = 95	*n* = 53	*n* = 23		*n* = 93	*n* = 78		*n* = 10	*n* = 55	*n* = 106	
median	4.94	4.93	4.85	6.55	0.777	4.93	5.18	5.48	0.811	3.77	8.40	0.013*,**	5.25	4.48	5.31	0.505
(range)	(0.07–7818.30)	(0.08–7818.30)	(0.21–333.40)	(0.07–193.94)		(0.07–7818.30)	(0.08–193.94)	(0.19–156.38)		(0.07–193.94)	(0.43–7818.30)		(2.17–179.05)	(0.07–7818.30)	(0.08–333.40)	
DICER1	*n* = 164	*n* = 93	*n* = 40	*n* = 31		*n* = 87	*n* = 54	*n* = 23		*n* = 89	*n* = 75		*n* = 11	*n* = 51	*n* = 102	
median	0.99	0.91	1.00	1.25	0.304	1.03	0.90	0.88	0.550	1.01	0.87	0.763	1.68	0.95	0.89	0.698
(range)	(0.21–119.51)	(0.24–119.51)	(0.21–7.76)	(0.25–5.07)		(0.21–45.80)	(0.25–119.51)	(0.27–5.70)		(0.21–7.76)	(0.24–119.51)		(0.27–2.23)	(0.21–45.80)	(0.25–119.51)	

*AC* adenocarcinoma, *SCC* squamous cell carcinoma, *p-value < 0.05 Mann–Whitney U test. **p-value < 0.05 Kruskal-Wallis test

### HMGA2 protein expression

**Fig. 2:**
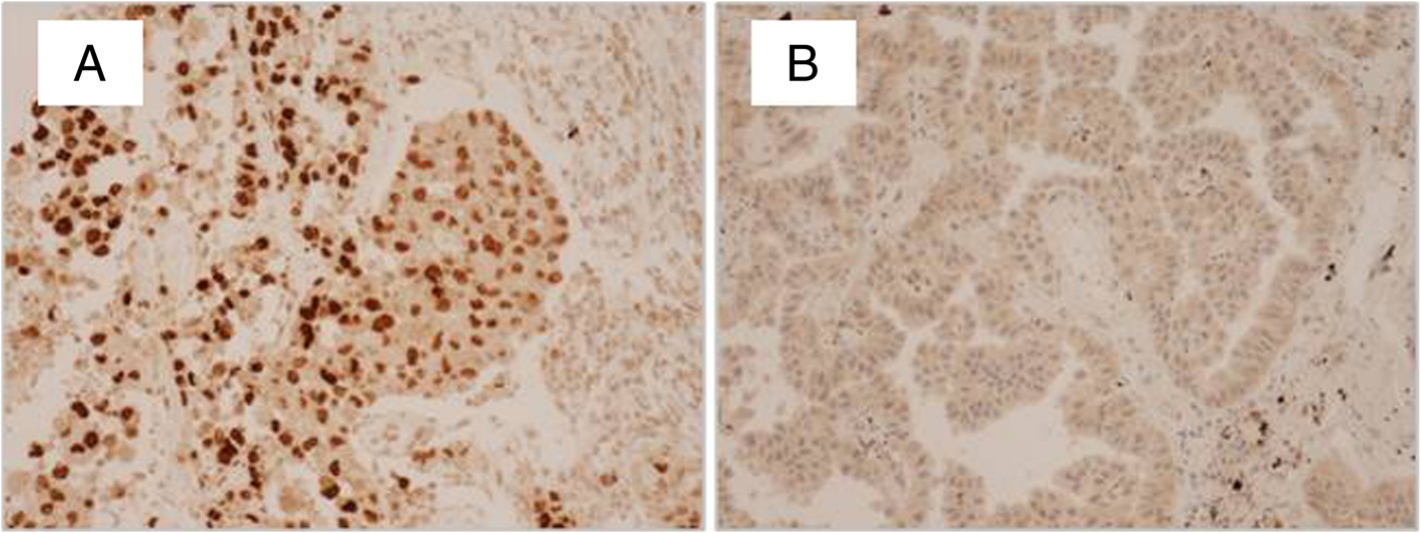
Expression of HMGA2 protein in NSCLC. Photomicrographs of NSCLC specimens stained with anti-HMGA2 antibodies. **a** represents a squamous cell carcinoma sample demonstrating strong immunoreactivity whereas negative staining is shown in **b**, an adenocarcinoma sample

In total, 86 (55 %) of the tumour samples were scored with a high expression level of the HMGA2 protein. In histological subsets, 90 % of the squamous cell carcinomas expressed high levels of HMGA2, while 47 % of the adenocarcinomas showed high expression of the protein; this difference was highly significant (*p* < 0.0001). It is worth noting that none of the 8 carcinoid tumours expressed HMGA2 protein. Protein expression levels according to histological subtypes are presented in Table 3. Microscopy images of HMGA2 protein in NSCLC are shown in Fig. 2.

**Table 3:** Levels of HMGA2 protein in histological subsets of non-small cell lung cancer

	Total	Low expression of HMGA2	Overexpression of HMGA2
Adenocarcinoma	88	47	41
Squamous cell carcinoma	38	4	34
Others	30	19	11
Total	156	70	86

### Correlations, mRNA expression and protein

A positive correlation coefficient of 0.513 was identified between *HMGA2* mRNA expression and HMGA2 protein expression (*p* < 0.010). *MYCN* and *HMGA2* mRNA expression showed a positive, albeit weaker correlation (correlation coefficient of 0.232, *p* < 0.010). *DICER1* mRNA expression was positively correlated to *MYCN*, *HMGA2* and *CDKN2A* mRNA expression with correlation coefficients of 0.463 (*p* < 0.010), 0.174 (*p* < 0.050) and 0.167 (*p* < 0.050) respectively (Additional file 1: Figure S3).

A significant correlation between *HMGA2* and *CDKN2A* mRNA was not seen. The correlation coefficient of *CDKN2A* mRNA expression and HMGA2 protein was weakly negative, however not significant.

### Let-7 microRNAs

All of the measured let-7 s had a mean expression level significantly lower in tumour tissue compared to normal lung tissue. Compared to tumours with low *HMGA2* mRNA expression, mean expression of *let-7a*, *let-7c*, *let-7d* and *let-7f* was significantly lower in tumours with high expression of *HMGA2* whereas *let-7d* was higher. Mean expression of *let-7a* and *let-7d* was significantly different depending on mRNA expression in all of the four genes in question, while mean *let-7f* was significantly different in *MYCN*, *HMGA2* and *CKDN2A* (Table 4). Details on the independent t-tests performed are included in supplementary tables (Additional file 1: Table S4, Additional file 1: Table S5, Additional file 1: Table S6, Additional file 1: Table S7, Additional file 1: Table S8).

**Table 4:** Mean let-7 microRNA expression associated with mRNA expression in tumour samples

	MYCN	HMGA2	CDKN2A	DICER1
Let-7a	0.044^*^	0.019^*^	0.025^*^	0.036^*^
Let-7a*	0.362^a^	0.207	0.720	0.265^a^
Let-7b	0.313	0.055	0.763	0.028^*^
Let-7c	0.729	0.021^*^	0.167	0.197
Let-7d	0.022^*^	0.038^*^	0.041^*^	0.040^*^
Let-7d*	0.107	0.301	0.040^*^	0.552
Let-7e	0.104	0.073	0.002^*^	0.102
Let-7f	0.048^*^	0.023^*^	0.019^*, a^	0.081
Let-7 g	0.012^*^	0.013^*, a^	0.030^*^	0.037^*^
Let-7i	0.166	0.105	0.097^a^	0.006^*^

Associations presented with *p*-values only. *p-value < 0.05, calculated by independent samples *t*-test. ^a^equal variance not assumed (Levene’s *F* test)

### Survival analyses

**Fig. 3:**
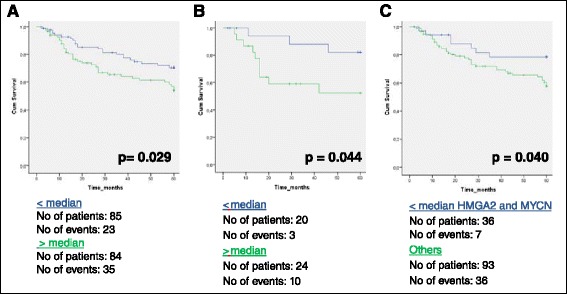
Associations between gene expression and patient outcome. mRNA fold change values dichotomized to high (*green*) or low (*blue*) expression based on the median expression level. High expression of *MYCN* mRNA had a significantly worse prognosis compared to lower expression levels in all tumour samples (**a**, *p* = 0.029) and in a subset of squamous cell carcinomas (**b**, *p* = 0.044). Lower expression of both *MYCN* and *HMGA2* (co-expressed) had a significantly better prognosis compared to the other alternatives (**c**, *p* = 0.040)

In survival analyses patients with a *MYCN* mRNA expression level above median had a significantly worse prognosis compared to patients with *MYCN* mRNA expression level below median, both in the cohort as a whole (log rank test, *p* = 0.029) and in patients with squamous cell carcinomas (log rank test, *p* = 0.044) (Fig. 3). In the multivariate Cox regression analysis adjusting for age, gender, stage and histology, the association between *MYCN* and time to recurrence was confirmed (*p* = 0.020) (Table 5). These findings were not, however, validated in a squamous cell carcinoma cohort with clinical follow-up data from the TCGA project or in the cohort with mRNA data from adenocarcinomas.

**Table 5:** Predictors for Progression Free Survival (*n* = 174)

Variable	Univariate Cox model			Multivariate Cox model		
	HR	95 % CI	*p*-value	HR	95 % CI	*p*-value
Age at surgery	1.01	0.98–1.04	0.713	1.01	0.98–1.04	0.428
Sex						
Male	Reference			Reference		
Female	1.38	0.83–2.3	0.218	1.46	0.85–2.51	0.170
Stage						
Stage I	Reference			Reference		
Stage II + III	1.36	0.82–2.26	0.234	1.26	0.75–2.12	0.389
Histology						
Adenocarcinoma	Reference			Reference		
Squamous cell carcinoma	0.94	0.50–1.78	0.850	1.06	0.55–2.07	0.856
Other	1.08	0.56–2.09	0.814	1.30	0.65–2.59	0.458
Chemotherapy						
Chemotherapy	Reference					
No chemotherapy	1.26	0.72–2.18	0.421			
Smoking status						
Current	Reference					
Former	1.22	0.71–2.09	0.478			
Never	1.33	0.52–3.40	0.557			
Tumor size						
≤ 3 cm	Reference					
> 3 cm - ≤ 5 cm	1.67	0.97–2.89	0.064			
> 5 cm	1.13	0.50–2.57	0.776			
EGFR status						
EGFR not mutated	Reference					
EGFR mutated	0.93	0.37–2.33	0.876			
Weight loss						
No weight loss	Reference					
< 10 %	1.28	0.70–2.39	0.422			
≥ 10 %	1.70	0.74–3.93	0.214			
MYCN						
*<* median expression	Reference					
*>* median expression	1.78	1.05–3.02	0.032*	1.90	1.11–3.26	0.020*
HMGA2						
*<* median expression	Reference					
*>* median expression	1.18	0.66–2.11	0.588			
*CDKN2A*						
*<* median expression	Reference					
> median expression	1.37	0.81–2.29	0.239			
DICER1						
*<* median expression	Reference					
*>* median expression	1.02	0.61–1.72	0.943			

mRNA expression data represented in the analysis as dichotomous variables (fold change). *p-value < 0.05

In tumours with apparent co-regulation of expression of *HMGA2* and *MYCN*, there was a significantly better outcome when both genes were downregulated compared to the alternatives (log rank test, *p* = 0.040) (Fig. 3).

Among patients with stage I disease, low HMGA2 protein expression was associated with better prognosis compared to the patients with overexpression of the protein (log rank test, *p* = 0.034) (Additional file 1: Figure S4). Patients with large cell carcinomas and low expression of the HMGA2 protein also had a better outcome (log rank test, *p* = 0.014). There was no significant relationship between HMGA2 protein expression and progression free survival among all patients included as a whole. Moreover, the mRNA levels of *CDKN2A* or *DICER* did not influence the prognosis in the different histological subgroups.

## Discussion

The development of NSCLC includes multiple genetic and epigenetic alterations that may lead to activation of pathways promoting tumour growth as well as inhibition of pathways of tumour suppression. NSCLC has among the greatest number of genetic aberrations of all malignant tumours [21]. This is the first study investigating the pathway involving genes *MYCN*, *HMGA2*, *CDKN2A*, *DICER1* and the let-7 family of microRNAs in NSCLC, while several findings have indicated a connected pathway involving these players [14–17].

The HMGA2 protein plays an important role in growth during embryonic development. It is mainly expressed in embryos, and mutant mice with HMGA2 deficiency develop a pygmy phenotype [22]. Close to absent in adult tissue, re-expression in tumours has led to investigations of causality. It is previously known that *HMGA2* chromosomal rearrangements are implicated in benign tumours of mesenchymal origin [23] and overexpression of the HMGA2 protein is found in a variety of malignant tumour types such as breast cancer [24], pancreatic cancer [25], oral squamous cell carcinomas [26] as well as NSCLC [6–8]. In our study, analysis of fresh-frozen NSCLC tumour tissue by RT-qPCR revealed a significant difference in *HMGA2* mRNA expression in histological subsets; with an elevated median expression value in the squamous cell carcinomas of approximately 5-fold over the median value of adenocarcinomas. A similar pattern was seen for HMGA2 protein; with a significant distinction of nuclear staining in the different histological groups. Close to all squamous cell carcinomas expressed high levels of HMGA2 compared to 46 % of the adenocarcinomas, and this difference was highly significant. Moreover, a strong positive correlation was demonstrated between *HMGA2* mRNA and HMGA2 protein expression. Previous studies have indicated a role for thyroid transcription factor-1 (TTF-1) in the regulation of HMGA2 expression, as a loss of TTF-1 triggers overexpression of HMGA2 [27]. Indeed, squamous cell carcinomas are usually negative for TTF-1, while the opposite is shown in adenocarcinomas [28].

An increase in *HMGA2* expression (RT-qPCR) in tumour matching non-tumour tissue, with a significant higher increase in squamous cell carcinoma compared to adenocarcinoma, was previously reported by Meyer et al. [6]. These results are consistent with our findings. Many studies highlight the role of HMGA2 protein in cancer progression, HMGA2 protein levels in primary lung tumours has been shown to correlate with increasing tumour grade and has furthermore been regarded as a necessity for a transformed phenotype in metastatic NSCLC cell lines [7]. Accordingly, HMGA2 protein expression was entirely devoid in the slow growing carcinoids in our study and HMGA2 protein levels in stage I patients were associated with progression free survival supporting the role of HMGA2 as a marker of aggressiveness.

We identified a positive correlation between *MYCN* and *HMGA2* mRNA. In addition, *DICER1* expression was positively correlated to *MYCN*, *HMGA2* and *CDKN2A*, supporting the notion of the involvement of this pathway. When focusing on the tumours with seemingly co-regulated *HMGA2* and *MYCN* expression, we found a significantly better outcome when both genes were downregulated compared to the remainders (Fig. 3). This also supports the involvement of this pathway in lung carcinomas, and has not been demonstrated previously. However, it is known in general that the genes investigated are also regulated by several other factors and that the contribution of HMGA2, in particular, is complex, involving several other genes and proteins [4].

Let-7 microRNA expression is scarce in embryonic stages, but increases in tissue following mature differentiation and is suggested as putative tumor suppressors [29]. As confirmed by our study, normal lung tissue showed a higher level of let-7 expression compared to adenocarcinoma tumor samples. Moreover, HMGA2 is a well characterized target of the let-7 microRNA family and known to be inversely correlated with HMGA2 expression in NSCLC cells [30, 31]. We found several let-7 microRNAs to be differentially expressed comparing high and low *HMGA2* gene expression; *let-7a*, *let-7c* and *let-7f* was seen inversely correlated, consistent with the biological presumption that loss of *let-7* inhibition leads to *HMGA2* overexpression in cancer. It has also been shown that aberrant expression of let-7 is more common in squamous cell carcinoma compared to adenocarcinomas [32]. Our cohort included only a few squamous cell carcinoma samples, but we still received a similar end result.

Although we identified a significant impact of *MYCN*-expression levels on progression free survival in both the cohort investigated as a whole, and in the squamous cell carcinomas alone, this was not validated in the TCGA data. There may be several explanations for this lack of validation. The TCGA project was initiated for molecular analyses, and the clinical data was missing in many of the available cases. Moreover, a fairly large proportion of patients with existing clinical data had only a short term follow-up. Our cohort in the present study consisted of several histological subgroups, and each subgroup had limited number of cases. We propose that similar survival analyses need to be further investigated in larger cohorts of homogenous subtypes in future studies.

The mRNA level of *HMGA2*, *CDKN2A* and *DICER1* did not influence survival in our study significantly. Previous studies have not investigated this fully in specific histological lung cancer subgroups, but have shown that the HMGA2 protein is involved in the transformation of lung cancer cells. Its role might therefore very well be in the initiation and establishment of the cancer. In neuroblastoma, *MYCN*, *LIN28B* and let-7 seem to be involved and *MYCN* amplification is correlated with adverse clinical outcome [33, 34]. This is a more benign disease although the location of tumours may unfavourably impact the prognosis.

One of the limitations to this study is the low number of samples in our subgroup analyses. In addition, cohorts with solid clinical data and follow up would ensure further investigations of the prognostic impact activation of this pathway in lung cancers, and in the different histological subtypes.

## Conclusion

In this study we have identified a significant difference between the histological subtypes of NSCLC and HMGA2 expression at both the mRNA and protein level. The more benign histology of carcinoids lacked HMGA2 expression, while among squamous cell carcinomas, most samples showed high expression. We have also demonstrated a correlation between mRNA expression of *HMGA2* and HMGA2 protein expression. The mRNA expression levels of *MYCN* and *HMGA2* and *DICER1* were significantly correlated, and co-regulation of expression of *HMGA2* and *MYCN*, had a significant impact on survival. The impact on survival is probably complex, and needs to be investigated in larger cohorts of specific histological non-small cell lung cancer subgroups.

## Additional file

Additional file 1: Table S1. Characteristics of the microRNA sample set. Table S2: Characteristics of the adenocarcinoma validation sample set. Table S3: Characteristics of the squamous cell carcinoma (TCGA) validation sample set. Table S4: Independent samples *t*-test. Mean let-7 microRNA expression in tumour and normal tissue samples. Table S5: Independent samples *t*-test. Mean let-7 microRNA expression and association with *MYCN* mRNA expression in tumour samples. Table S6: Independent samples *t*-test. Mean let-7 microRNA expression and association with *HMGA2* mRNA expression in tumour samples. Table S7: Independent samples *t*-test. Mean let-7 microRNA expression and association with *CDKN2A* mRNA expression in tumour samples. Table S8: Independent samples *t*-test. Mean let-7 microRNA expression and association with *DICER1* mRNA expression in tumour samples and additional figures (Figure S1: REMARK diagram detailing sample availability and use of different analytical techniques in the present study. Figure S2: Boxplots illustrating the distribution of gene expression (fold change) from the RT-qPCR . Figure S3: Scatterplots illustrating significant correlations. Figure S4: Associations between HMGA2 protein expression and patient outcome). Figure S1. REMARK diagram detailing sample availability and use of different analytical techniques in the present study. Figure S2. Boxplots illustrating the distribution of gene expression from the RT-qPCR. Figure S3. Scatterplots illustrating significant correlations. Figure S4. Associations between NSCLC stage, HMGA2 protein expression and patient outcome. HMGA2 protein expression values dichotomized to low expression (blue) and overexpression (green) based on immunohistochemistry. Low expression of HMGA2 protein had a significantly better prognosis compared to overexpression in stage I non-small cell lung cancer tumour samples (A, *p* = 0.034). No significant association in stage II (B, *p* = 0.492) or stage III (C, *p* = 0.862) patients was seen. (DOCX 401 kb)

## Abbreviations

ACTB: Actin-beta
ALK: Anaplastic lymphoma kinase
CDKN2A: Cyclin-dependent kinase inhibitor 2A
EGFR: Epidermal growth factor receptor
HMGA2: High mobility group AT-hook 2
MYCN: V-myc avian myelocytomatosis viral oncogene neuroblastoma derived homolog
NSCLC: Non-small cell lung cancer
RT-qPCR: Reverse transcription quantitative polymerase chain reaction
TCGA: The Cancer Genome Atlas
TMA: Tissue micro array
TNM: TNM classification of malignant tumours
TTF-1: Thyroid transcription factor-1
UICC: Union for International Cancer Control
