# Supplementary material for: The MYCN-HMGA2-CDKN2A pathway in non-small cell lung carcinoma—differences in histological subtypes
Source: BMC Cancer. 2016 Feb 8;16:71. doi: 10.1186/s12885-016-2104-9 (PMC4746877; doi:10.1186/s12885-016-2104-9)
Supplement: Additional file 1: Table S1. — Characteristics of the microRNA sample set. Table S2: Characteristics of the adenocarcinoma validation sample set. Table S3: Characteristics of the squamous cell carcinoma (TCGA) validation sample set. Table S4: Independent samples t-test. Mean let-7 microRNA expression in tumour and normal tissue samples. Table S5: Independent samples t-test. Mean let-7 microRNA expression and association with MYCN mRNA expression in tumour samples. Table S6: Independent samples t-test. Mean let-7 microRNA expression and association with HMGA2 mRNA expression in tumour samples. Table S7: Independent samples t-test. Mean let-7 microRNA expression and association with CDKN2A mRNA expression in tumour samples. Table S8: Independent samples t-test. Mean let-7 microRNA expression and association with DICER1 mRNA expression in tumour samples and additional figures (Figure S1: REMARK diagram detailing sample availability and use of different analytical techniques in the present study. Figure S2: Boxplots illustrating the distribution of gene expression (fold change) from the RT-qPCR . Figure S3: Scatterplots illustrating significant correlations. Figure S4: Associations between HMGA2 protein expression and patient outcome). Figure S1. REMARK diagram detailing sample availability and use of different analytical techniques in the present study. Figure S2. Boxplots illustrating the distribution of gene expression from the RT-qPCR. Figure S3. Scatterplots illustrating significant correlations. Figure S4. Associations between NSCLC stage, HMGA2 protein expression and patient outcome. HMGA2 protein expression values dichotomized to low expression (blue) and overexpression (green) based on immunohistochemistry. Low expression of HMGA2 protein had a significantly better prognosis compared to overexpression in stage I non-small cell lung cancer tumour samples (A, p = 0.034). No significant association in stage II (B, p = 0.492) or stage III (C, p = 0.862) patients was seen. (DOCX 401 kb) [file 12885_2016_2104_MOESM1_ESM.docx]

**Additional files:**


**Table S1: Characteristics of the microRNA sample set**

| Variable: | Patients:  No: 78 | % |
| --- | --- | --- |
| *Sex:* |  |  |
| Male: | 38 | 48.7 |
| Female: | 40 | 51.3 |
| *Smoking history:* |  |  |
| Current: | 41 | 52.6 |
| Former:  Never: | 29  8 | 37.2  10.3 |
| *Stage:* |  |  |
| I: | 46 | 59.0 |
| II: | 18 | 23.1 |
| IIIa: | 14 | 17.9 |
| *Histology:* |  |  |
| Adenocarcinoma: | 75 | 96.2 |
| Squamous cell carcinoma: | 2 | 2.6 |
| Other: | 1 | 1.3 |
| *EGFR:* |  |  |
| EGFR mutated: | 11 | 14.1 |
| EGFR not mutated: | 67 | 85.9 |
| *Status per 31.01.2015:* |  |  |
| Alive: |  |  |
| Alive with no disease: | 42 | 53.8 |
| Alive with disease: | 9 | 11.5 |
| Dead: | 27 | 34.7 |

**Table S2: Characteristics of the adenocarcinoma validation sample set**

| Variable: | Patients: |  |
| --- | --- | --- |
| *Age(years):* |  |  |
| Mean: | 66.27 |  |
| Median: | 66.20 |  |
| Range: | 39-87 |  |
|  |  |  |
|  | No: 187 | % |
| *Sex:* |  |  |
| Male: | 82 | 43.9 |
| Female: | 105 | 56.1 |
| *Stage:* |  |  |
| I: | 112 | 59.9 |
| II: | 41 | 21.9 |
| IIIa: | 34 | 18.2 |
| *Histology:* |  |  |
| Adenocarcinoma: | 182 | 97.3 |
| Squamous cell carcinoma: | 5 | 2.7 |
| *EGFR:* |  |  |
| EGFR mutated: | 23 | 12.3 |
| EGFR not mutated: | 161 | 86.1 |
| EGFR not tested | 3 | 1.6 |
| *Smoking history:* |  |  |
| Current: | 63 | 33.7 |
| Former: | 103 | 55.1 |
| Never: | 21 | 11.2 |
| *PFS, event during follow-up:* |  |  |
| Yes: | 74 | 39.6 |
| No: | 113 | 60.4 |

**Table S3: Characteristics of the squamous cell carcinoma (TCGA) validation sample set**

| Variable: | Patients: |  |
| --- | --- | --- |
| *Age(years):* |  |  |
| Mean: | 67.7 |  |
| Median: | 69 |  |
| Range: | 39-90 |  |
|  |  |  |
|  | No: 280 | % |
| *Gender:* |  |  |
| Male: | 194 | 69.3 |
| Female: | 86 | 30.7 |
| *Stage:* |  |  |
| I: | 140 | 50.0 |
| II: | 98 | 35.0 |
| IIIa: | 42 | 15.0 |
| *Histology:* |  |  |
| Squamous cell carcinoma: | 280 | 100 |
| *EGFR:* |  |  |
| EGFR mutated: | 19 | 6.8 |
| EGFR not mutated: | 181 | 64.6 |
| EGFR not tested | 80 | 28.6 |
| *ALK:* |  |  |
| ALK positive | 9 | 3.2 |
| ALK negative | 180 | 64.3 |
| ALK not tested | 91 | 32.5 |
| *Smoking history:* |  |  |
| Current: | 79 | 28.2 |
| Former: | 189 | 67.5 |
| Never: | 6 | 2.1 |
| Missing data: | 6 | 2.1 |
| *PFS, event during follow-up:* |  |  |
| Yes: | 76 | 27.1 |
| No: | 204 | 72.9 |

**Table S4: Independent samples t-test. Mean let-7 microRNA expression in tumour and normal tissue samples.**

|  |  | n | mean | SD | t | df | p |
| --- | --- | --- | --- | --- | --- | --- | --- |
| Let-7a | Tumor  Normal | 16  16 | -0.48  0.82 | 0.76  0.28 | -6.45 | 18.98 | 0.000*† |
| Let-7a* | Tumor  Normal | 16  16 | 0.91  3.18 | 2.39  1.82 | -3.03 | 30 | 0.005* |
| Let-7b | Tumor  Normal | 16  16 | 0.18  0.90 | 0.44  0.31 | -5.29 | 30 | 0.000* |
| Let-7c | Tumor  Normal | 16  16 | 0.05  1.09 | 0.55  0.30 | -6.69 | 30 | 0.000* |
| Let-7d | Tumor  Normal | 16  16 | -0.57  -0.69 | 0.71  0.30 | -6.56 | 20.29 | 0.000*† |
| Let-7d* | Tumor  Normal | 16  16 | -0.10  2.43 | 0.43  2.09 | -4.74 | 16.25 | 0.000*† |
| Let-7e | Tumor  Normal | 16  16 | -0.63  0.50 | 0.81  0.31 | -5.22 | 19.29 | 0.000*† |
| Let-7f | Tumor  Normal | 16  16 | -0.69  0.74 | 0.91  0.29 | -6.00 | 18.14 | 0.000*† |
| Let-7g | Tumor  Normal | 16  16 | -0.66  0.58 | 0.91  0.30 | -5.21 | 18.21 | 0.000*† |
| Let-7i | Tumor  Normal | 16  16 | -0.45  -0.00 | 0.68  0.31 | -2.41 | 21.05 | 0.025*† |

*p-value < 0.05 †equal variance not assumed (Levene`s *F* test)

**Table S5: Independent samples t-test. Mean let-7 microRNA expression and association with MYCN mRNA expression in tumour samples**

|  | MYCN  Fold change | n | mean | SD | t | df | P |
| --- | --- | --- | --- | --- | --- | --- | --- |
| Let-7a | < median  > median | 29  44 | 0.00  -0.43 | 0.74  0.95 | 2.05 | 71 | 0.044* |
| Let-7a* | < median  > median | 29  44 | 0.60  0.99 | 1.60  2.04 | -0.92 | 68.67 | 0.362† |
| Let-7b | < median  > median | 29  44 | 0.01  -0.12 | 0.44  0.57 | 1.02 | 71 | 0.313 |
| Let-7c | < median  > median | 29  44 | -0.17  -0.12 | 0.54  0.68 | -0.35 | 71 | 0.729 |
| Let-7d | < median  > median | 29  44 | -0.02  -0.47 | 0.67  0.89 | 2.34 | 71 | 0.022* |
| Let-7d* | < median  > median | 29  44 | 0.16  -0.11 | 0.66  0.70 | 1.63 | 71 | 0.107 |
| Let-7e | < median  > median | 29  44 | -0.13  -0.54 | 0.77  1.17 | 1.65 | 71 | 0.104 |
| Let-7f | < median  > median | 29  44 | -0.01  -0.50 | 0.82  1.14 | 2.01 | 71 | 0.048* |
| Let-7g | < median  > median | 29  44 | 0.06  -0.51 | 0.74  1.04 | 2.58 | 71 | 0.012* |
| Let-7i | < median  > median | 29  44 | 0.06  -0.21 | 0.72  0.87 | 1.40 | 71 | 0.166 |

*p-value < 0.05 †equal variance not assumed (Levene`s *F* test)

**Table S6: Independent samples t-test. Mean let-7 microRNA expression and association with HMGA2 mRNA expression in tumour samples**

|  | HMGA2  Fold change | n | mean | SD | t | df | P |
| --- | --- | --- | --- | --- | --- | --- | --- |
| Let-7a | < median  > median | 28  27 | 0.00  -0.66 | 0.72  1.21 | 2.41 | 53 | 0.019***** |
| Let-7a* | < median  > median | 28  27 | 1.16  0.48 | 2.02  1.21 | 1.28 | 53 | 0.207 |
| Let-7b | < median  > median | 28  27 | 0.08  -0.24 | 0.51  0.13 | 1.97 | 53 | 0.055 |
| Let-7c | < median  > median | 28  27 | 0.09  -0.38 | 0.60  0.83 | 2.37 | 53 | 0.021* |
| Let-7d | < median  > median | 28  27 | -1.11  -0.64 | 0.61  1.16 | 2.13 | 53 | 0.038* |
| Let-7d* | < median  > median | 28  27 | -0.02  -0.19 | 0.61  1.16 | 1.04 | 53 | 0.301 |
| Let-7e | < median  > median | 28  27 | -0.18  -0.69 | 0.79  1.26 | 1.83 | 53 | 0.073 |
| Let-7f | < median  > median | 28  27 | -0.05  -0.76 | 0.81  1.36 | 2.34 | 53 | 0.023***** |
| Let-7g | < median  > median | 28  27 | -0.51  -0.71 | 0.66  1.16 | 2.58 | 41.00 | 0.013*† |
| Let-7i | < median  > median | 28  27 | 0.03  -0.28 | 0.67  0.76 | 1.65 | 53 | 0.105 |

*p-value < 0.05 †equal variance not assumed (Levene`s *F* test)

**Table S7: Independent samples t-test. Mean let-7 microRNA expression and association with CDKN2A mRNA expression in tumour samples**

|  | MYCN  Fold change | n | mean | SD | t | df | P |
| --- | --- | --- | --- | --- | --- | --- | --- |
| Let-7a | < median  > median | 39  36 | -0.04  -0.58 | 0.79  1.23 | 2.28 | 73 | 0.025* |
| Let-7a* | < median  > median | 39  36 | 0.70  0.86 | 1.83  1.92 | -0.36 | 73 | 0.720 |
| Let-7b | < median  > median | 39  36 | -0.08  -0.12 | 0.51  0.64 | 0.30 | 73 | 0.763 |
| Let-7c | < median  > median | 39  36 | -0.06  -0.29 | 0.60  0.80 | 1.40 | 73 | 0.167 |
| Let-7d | < median  > median | 39  36 | -0.10  -0.56 | 0.80  1.09 | 2.08 | 73 | 0.041* |
| Let-7d* | < median  > median | 39  36 | -0.02  0.13 | 0.76  0.60 | -2.09 | 73 | 0.040* |
| Let-7e | < median  > median | 39  36 | -0.02  -0.85 | 0.84  1.34 | 2.26 | 73 | 0.002* |
| Let-7f | < median  > median | 39  36 | -0.03  -0.69 | 0.87  1.41 | 2.41 | 57.15 | 0.019*****† |
| Let-7g | < median  > median | 39  36 | -0.06  -0.57 | 0.83  1.18 | 2.21 | 73 | 0.030* |
| Let-7i | < median  > median | 39  36 | 0.09  -0.23 | 0.58  0.98 | 1.69 | 56.01 | 0.097† |

*p-value < 0.05 †equal variance not assumed (Levene`s *F* test)

**Table S8: Independent samples t-test. Mean let-7 microRNA expression and association with DICER1 mRNA expression in tumor samples**

|  | HMGA2  Fold change | n | mean | SD | t | df | p |
| --- | --- | --- | --- | --- | --- | --- | --- |
| Let-7a | < median  > median | 34  38 | -0.04  -0.57 | 1.17  0.93 | 2.13 | 70 | 0.036* |
| Let-7a* | < median  > median | 34  38 | 0.52  1.01 | 1.60  2.05 | -1.13 | 66.81 | 0.265† |
| Let-7b | < median  > median | 34  38 | 0.06  -0.24 | 0.59  0.56 | 2.24 | 70 | 0.028* |
| Let-7c | < median  > median | 34  38 | -0.06  -0.28 | 0.80  0.65 | 1.30 | 70 | 0.197 |
| Let-7d | < median  > median | 34  38 | -0.10  -0.58 | 1.06  0.87 | 2.10 | 70 | 0.040* |
| Let-7d* | < median  > median | 34  38 | 0.05  -0.05 | 0.75  0.67 | 0.60 | 70 | 0.552 |
| Let-7e | < median  > median | 34  38 | -0.19  -0.65 | 1.17  1.19 | 1.66 | 70 | 0.102 |
| Let-7f | < median  > median | 34  38 | -0.12  -0.62 | 1.29  1.12 | 1.77 | 70 | 0.081 |
| Let-7g | < median  > median | 34  38 | -0.05  -0.59 | 1.08  0.99 | 2.12 | 70 | 0.037* |
| Let-7i | < median  > median | 34  38 | 0.19  -0.34 | 0.73  0.83 | 2.86 | 70 | 0.006* |

*p-value < 0.05 †equal variance not assumed (Levene`s *F* test)

Figure S1. REMARK diagram detailing sample availability and use of different analytical techniques in the present study.


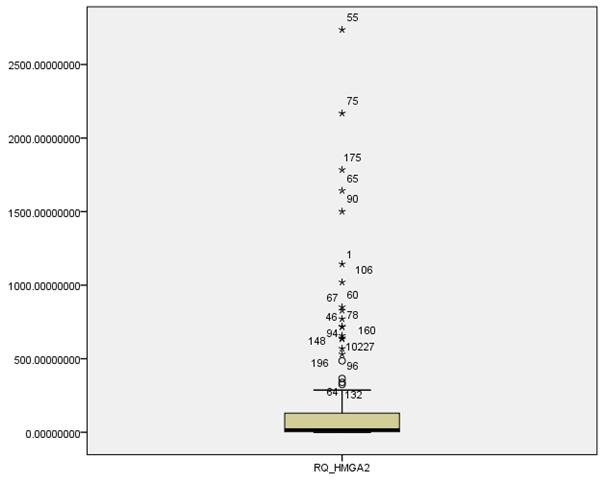

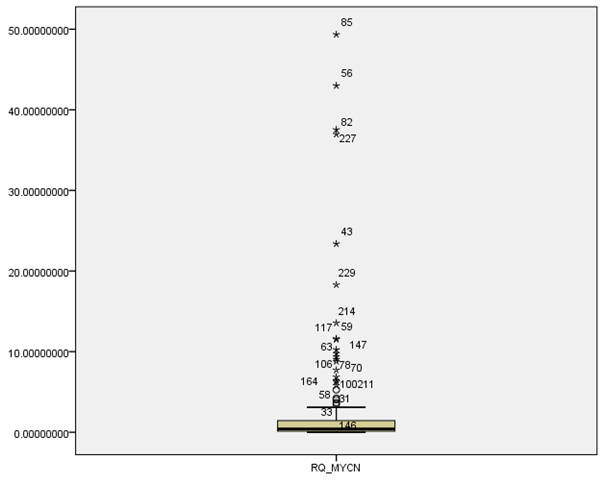


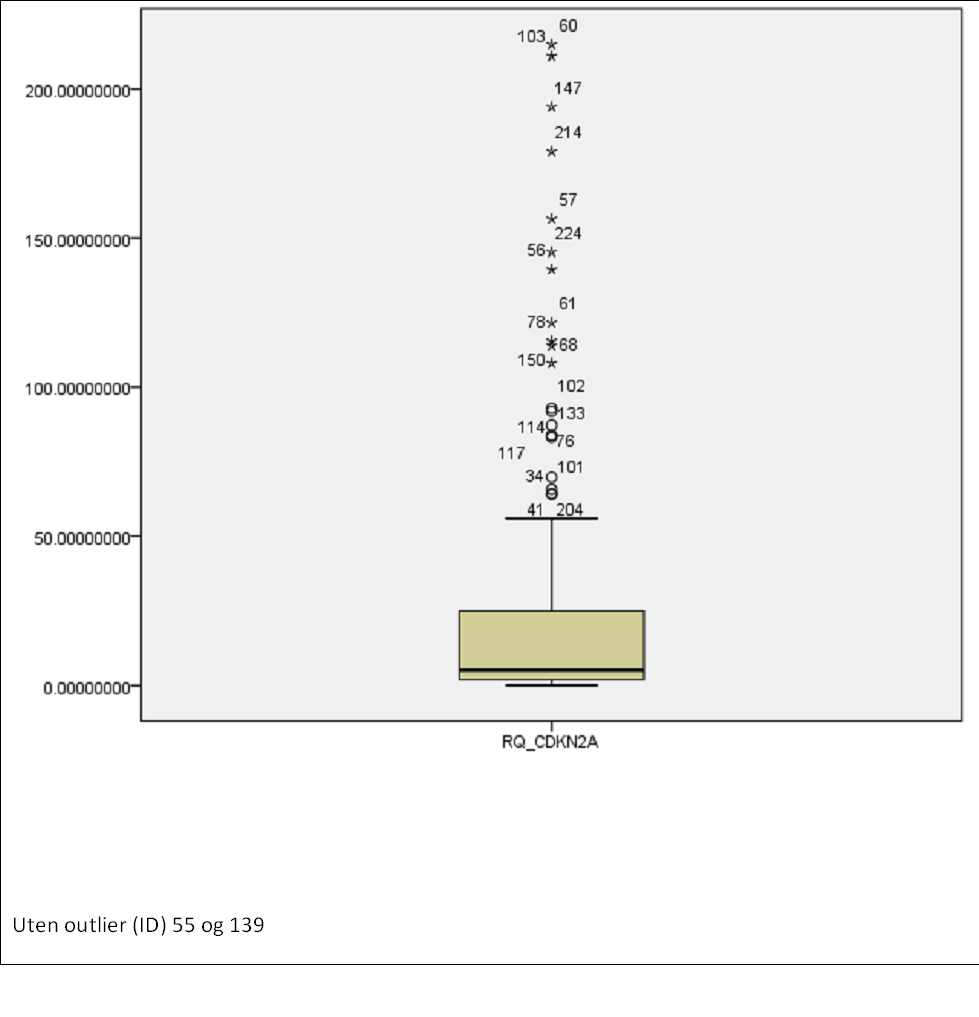

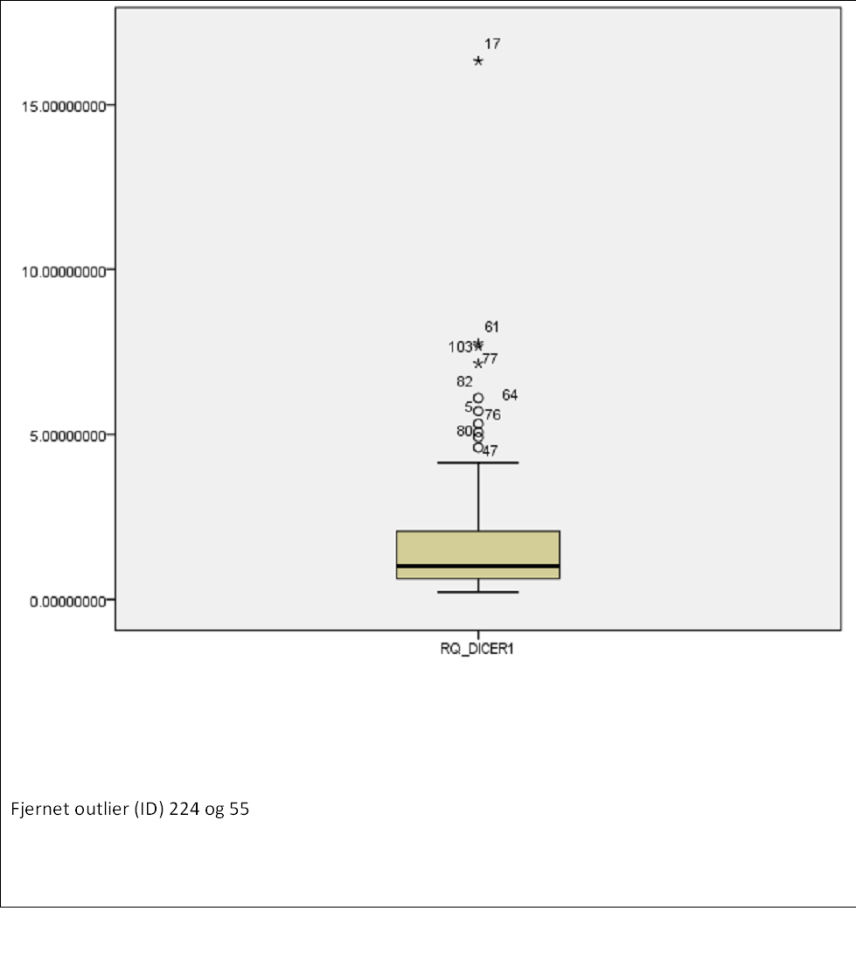


Figure S2. Boxplots illustrating the distribution of gene expression (fold change) from the RT-qPCR.


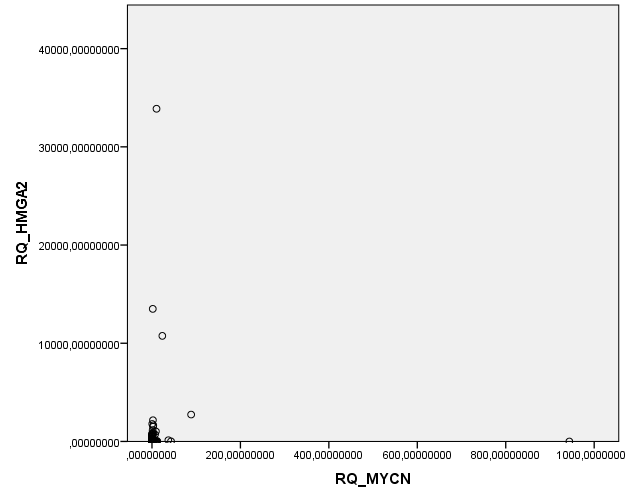

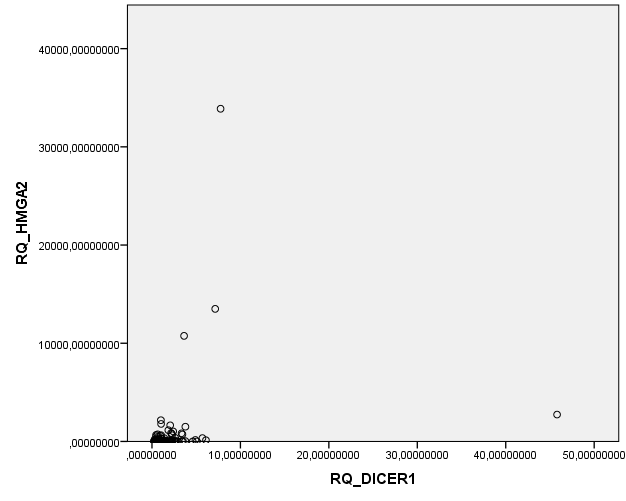


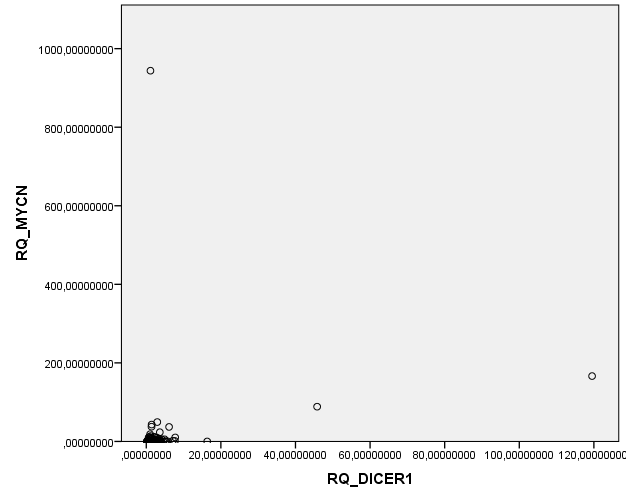

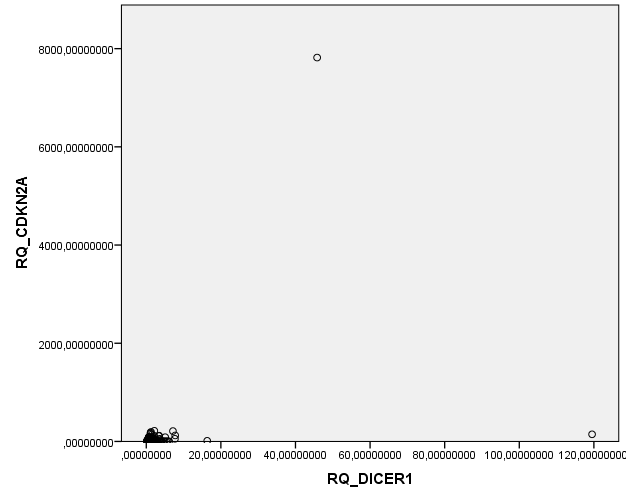


Figure S3. Scatterplots illustrating significant correlations.

**A B**

**
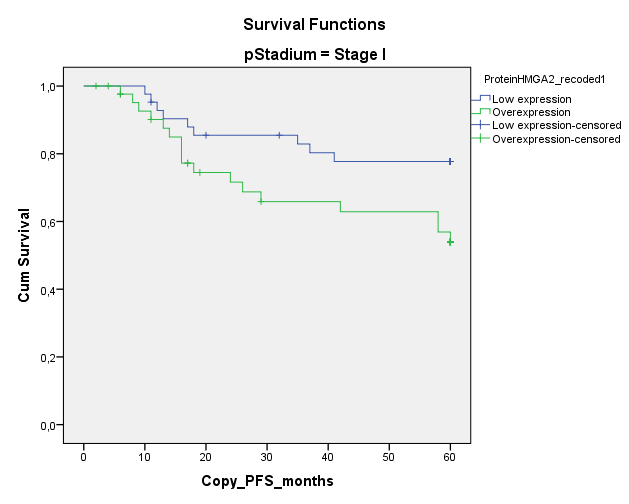
**  **
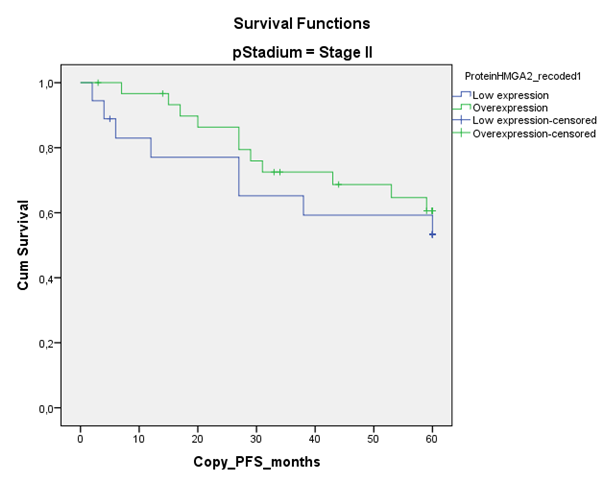
**

**Time (months)**

**Time (months)**

**p = 0.034**

**p = 0.492**

**C**

**
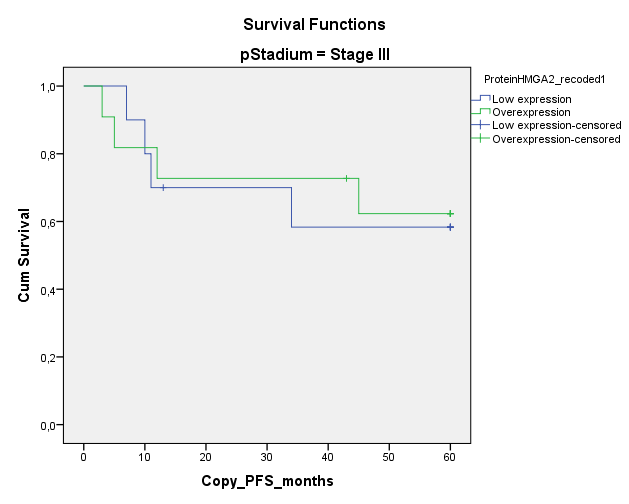
**

**Time (months)**

**p = 0.862**

**Figure S4.** **Associations between HMGA2 protein expression and patient outcome in different disease stage.** HMGA2 protein expression values dichotomized to low expression (blue) and overexpression (green) based on immunohistochemistry. Low expression of HMGA2 protein had a significantly better prognosis compared to overexpression in stage I non-small cell lung cancer tumour samples (A, p = 0.034). No significant association in stage II (B, p = 0.492) or stage III (C, p = 0.862) patients was seen.
